# A case and literature review of intraocular echinococcus causing bilateral visual loss in a HIV-infected patient

**DOI:** 10.1177/2050313X221113699

**Published:** 2022-08-28

**Authors:** Kylie Divashnee Konar, Somasundrum Pillay

**Affiliations:** 1 Phoenix Community Health Centre, Phoenix, South Africa; 2 Department of Internal Medicine, King Edward VIII Hospital (KEH), Durban, South Africa

**Keywords:** Echinococcosis, parasitic disease, intraocular, South Africa, hydatid disease

## Abstract

Although echinococcosis is a common parasitic disease endemic to Africa, ocular echinococcosis is rare. We present a case of intraocular echinococcosis in a HIV-infected patient. A 38-year-old South African HIV-infected woman presented to a district-level hospital in Kwa-Zulu Natal on 10 March 2020. Her main presenting complaint was that of progressive, painless, bilateral visual loss. A B-ultrasonography scan revealed tractional retinal detachment on the right eye, while the left eye had tractional bands with a ‘double-walled’ cystic cavity causing retinal traction. A systemic work-up revealed a positive Echinococcus ELISA IgG with a value of 1.3, eosinophilia of 5.70% (0.41 × 10^9^), and elevated C-reactive protein and erythrocyte sedimentation rate of 47 mg/L and 93 mm/hr, respectively. Based on the above clinical, biochemical and ultrasonographical evidence, a diagnosis of ocular echinococcosis was made. Our differential diagnosis includes toxic optic neuropathy, Jarisch-Herxheimer-like reaction secondary to immune reconstitution and necrotizing herpetic retinitis. She was initiated on topical and intravitreal steroids which led to decreased intraocular inflammation and dry maculae. Five months after presentation, her visual acuity remained unchanged with no light perception in both eyes. We conclude that ocular echinococcosis, although rare, can lead to severe visual impairment as there are no known definite treatment modalities for intraocular hydatid disease. Reports on co-infections with HIV and *Echinococcus* are limited with a potential scope for research.

## Introduction

Hydatid cystic disease in humans is caused by infestation with *Echinococcus granulosis*.^1^ Case reports on co-infections with HIV and *E. granulosis* are limited.^2,3^ The epidemiology of cystic echinococcosis in South Africa is unknown.^4^ Wahlers et al.^4^ found in their study of human echinococcosis that at least 137 cases are found annually in South Africa. The organism gains entry through the gut and spreads haematologically to various organ systems. The most common sites of the disease are the liver (50%–77%), lungs (18%–35%), abdominal cavity and brain.^5^ Ocular echinococcosis is rare, and most of the cases involve the orbit.^6–8^ Orbital involvement occurs in 1%–2% of hydatid disease cases.^5^ We report a case of intraocular echinococcosis causing visual loss in a HIV-infected patient.

## Case presentation

A 38-year-old South African woman presented to a district hospital in Durban, Kwa-Zulu Natal with progressive, painless bilateral visual loss since 10 March 2020. She had associated mild, intermittent headaches which were relieved by simple analgesia. Systemic enquiry was non-contributory.

She was diagnosed with HIV infection in 2006 and began antiretroviral treatment (ART) in 2012. She was switched to second-line ART in 2020 after reports of virological failure secondary to default. She was seen initially at McCord’s Provincial Eye Hospital (MPEH) on 10 March 2020 with a history of progressive loss of vision. She was noted to have bilateral panuveitis with macula oedema and disc pallor with non-perfused vessels. She was given topical and sub-Tenon’s steroids as well as bilateral bevacizumab (Avastin) injections for the macula oedema, which resolved. Her viral load at presentation was 208,806 copies/mL (15 January 2020), which worsened to 237,000 copies/mL (15 March 2020) after which she was switched to second-line ART as a result of treatment default. Her cluster of differentiation (CD4) count on this presentation was 39 cells/µL.

She was diagnosed with pulmonary tuberculosis (PTB) confirmed by typical chest radiographic changes and initiated on anti-TB treatment on 18 April 2020 after which she noticed worsening of the loss of vision in both eyes. She was then seen at MPEH on 25 May 2020 and was noted to have no perception of light (NPL) in both eyes. Fundus examination was still possible at this stage, which noted resolving macula oedema and disc pallor with non-perfused vessels.

She was admitted to a district hospital in Durban for vision loss in February 2021. She reported compliance to her ART, and on admission her HIV viral load was 136 copies/mL, while her CD4 count was 132 cells/µL. She did not report any history of eye trauma. Her surgical, ophthalmological, and family histories were otherwise unremarkable.

She had sober habits with a history of swimming in rivers. She was previously employed as a domestic worker with no history of toxic exposures and reported to have stayed on a farm and consumed cooked meat.

General examination revealed an obese, middle-aged woman. Her vital signs, including blood pressure, were within normal limits. On examination, her Snellen Visual Acuity (VA) was measured as being 6/60 in the right eye and 1/60 in the left eye with no improvement with pinhole testing. Anterior segment examination revealed bilateral corneal pigmented keratic precipitates with cells in the anterior chamber and anterior vitreous humour and lenticular opacities. Fundus examination revealed bilateral optic disc pallor with striae and exudates at the macula and retinal vasculitis with extensive non-perfused vessels.

**Figure 1.**
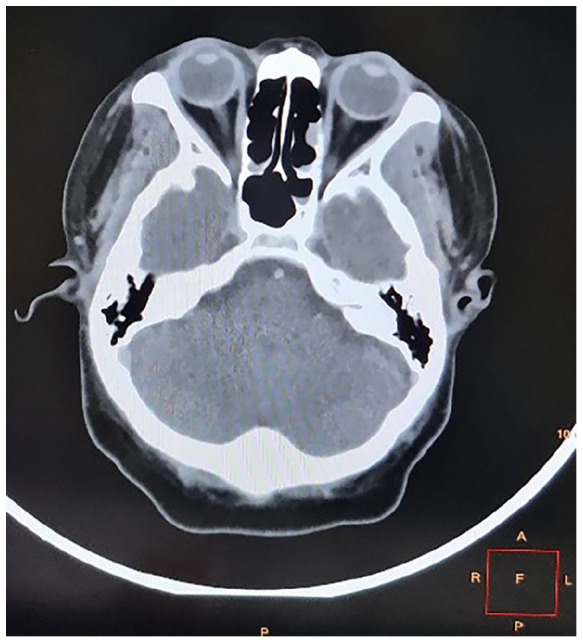
Axial section of brain. Figure 1 was found to have a focal irregular area of nodular non-enhancing soft tissue thickening in relation to the retina just adjacent to the optic nerve insertion, which appears hyperdense in the unenhanced study. This is suggestive of a retinal lesion (inflammatory, non-benign or haemorrhage) or artefactual. No calcifications are present. Intraocular and extracoronal spaces are intact bilaterally. Computed tomography (CT) image of patient’s brain.

**Figure 2.**
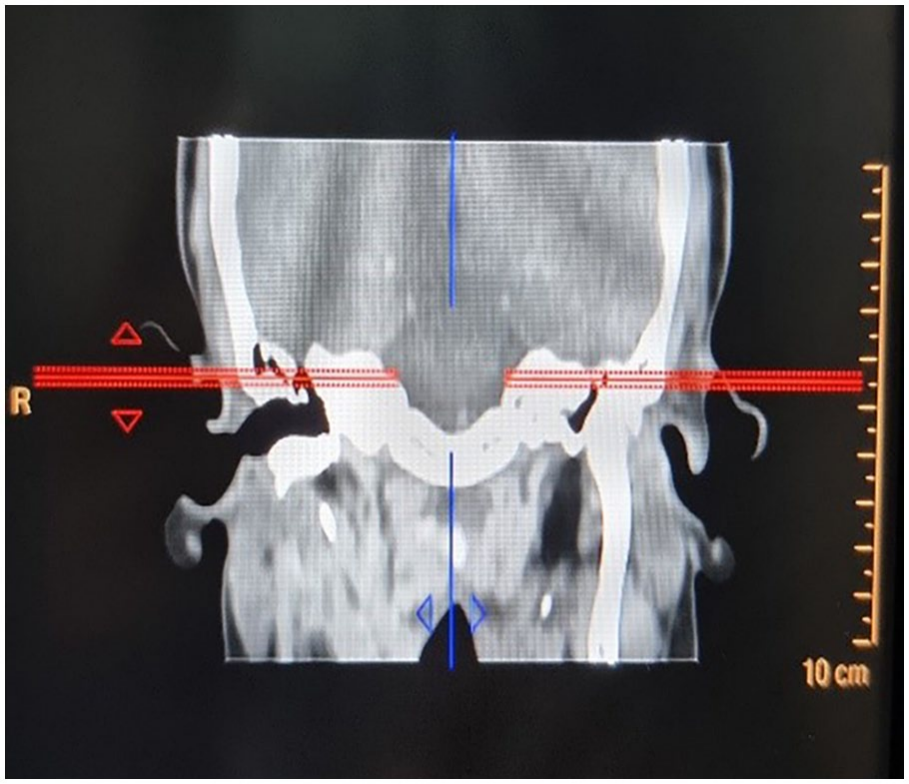
Coronal section of brain. Computed tomography (CT) image of patient’s brain.

**Figure 3.**
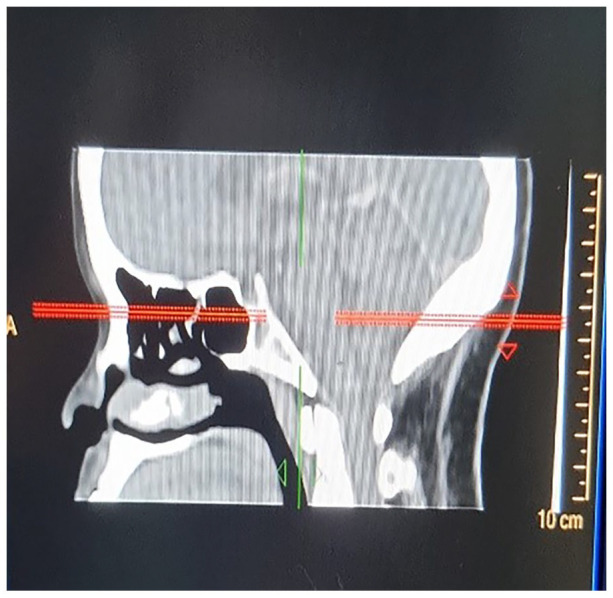
Lateral section of brain. Computed tomography (CT) image of patient’s brain.

The chest radiograph was normal. A computed tomography (CT) scan of the brain illustrated a small focus of nodular irregular thickening of the right retina just adjacent to the insertion of the optic nerve suggestive of an inflammatory lesion, non-benign lesion, haemorrhage or artefact. Clinically correlation was advised. There were no periventricular plaques, calcifications, infarcts, or pathological enhancements (Figures 1–3).

**Figure 4.**
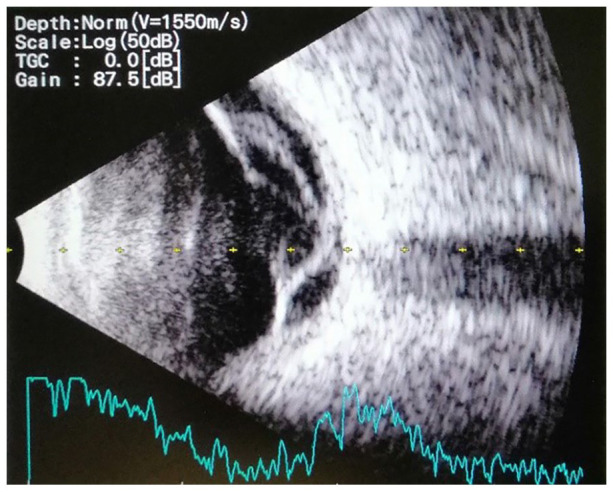
Right eye with tractional retinal detachment. Scan of B-ultrasonography of patients orbits.

**Figure 5.**
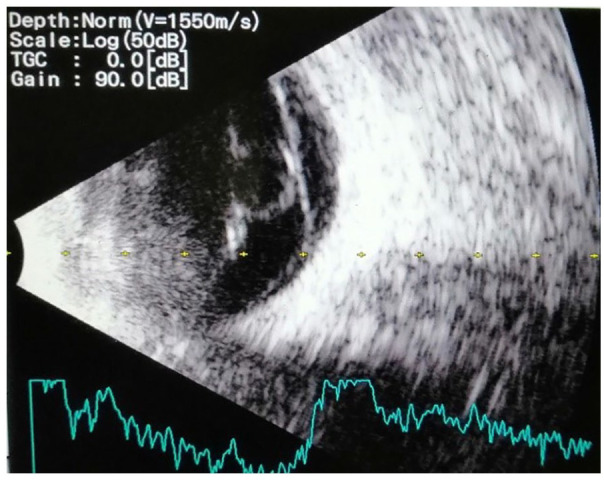
Left eye with tractional bands and a round intravitreal lesion. Scan of B-ultrasonography of patients orbits.

Figure 4 shows tractional retinal detachment on the right eye. Figure 5 depicts the left eye with tractional bands and shows a ‘double-walled sign’ suggestive of echinococcal cystic cavity, causing traction on the retina.^9^

A full systemic work-up was performed to exclude systemic echinococcus and other infections. A lumbar puncture was within normal range (Table 1). Liver ultrasound revealed no cystic lesions.

**Table 1.** Cerebrospinal fluid composition of the patient.

Test	Result	Reference range
Opening pressure	12 cm H_2_O	10–20 cm H_2_O
Cerebrospinal chemistry		
Glucose	3.2	60%–80% of plasma glucose
Protein	0.31	0.15–0.45
Microscopy		
Appearance	Clear, colourless, no clots	
Cell count		
Polymorphs	0	
Lymphocytes	2	
Erythrocytes	0	
Gram stain	No bacteria observed	
Culture	No growth after 3 days	
Adenosine deaminase	1.0 U/L	0–6
Herpes simplex virus 1 and 2 PCR	Negative	
Varicella zoster virus PCR	Negative	
Cytomegalovirus PCR	Negative	
Mycobacterium tuberculosis GeneXpert	Negative	
Venereal Disease Research Laboratory Test (VDRL)	Non-reactive	
Cryptococcal antigen test	Negative	

PCR: polymerase chain reaction.

Parasitic serology revealed a cysticercosis ELISA IgG negative value of 0.2, while the Echinococcus ELISA IgG was positive with a value of 1.3.

The full blood count revealed a white cell count of 7.26 × 10^9^ with an eosinophilia of 5.70% (0.41 × 10^9^). Her inflammatory markers, both C-reactive protein (CRP) and ertythrocyte sedimentation rate (ESR), were elevated at 47 mg/L and 93mm/hr, respectively. Non-specific markers in the form of Serum Angiotensin Converting Enzyme (SACE) and Rheumatoid Factor (RF) were weakly positive, at 101 U/L (8–52) and 13.0 IU/mL, respectively. Anti-nuclear antibodies and anti-nuclear factor were negative. Hepatitis screen was negative. Vitamin B12 and folate levels were normal.

Syphilis serology tests were negative (*Treponema pallidum* haemagglutinin assay, fluorescent treponemal antibody absorption test). Herpes simplex virus (HSV) IgM result was negative. Both cytomegalovirus IgM result and *Toxoplasma gondii* IgM ELISA result were negative. Anaerobic and aerobic blood cultures showed no growth. The rest of the blood parameters were within normal limits.

## Management and outcome

She was initiated on topical steroids and given bilateral orbital floor intravitreal steroid injections which resulted in decreased intraocular inflammation and dry maculae.

**Figure 6.**
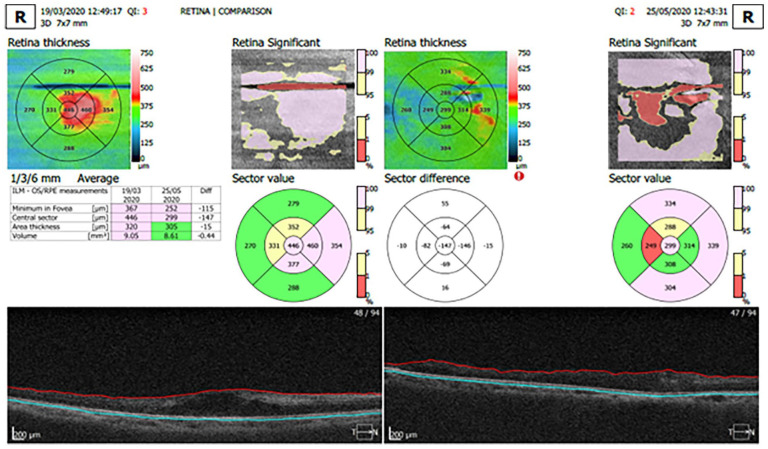
Optical coherence tomography (OCT) scan pre- and post-injection of right eye with orbital floor steroids. Significant decrease in inflammation was noted after injection of the right eye 2 months after steroid injection in 2020.

Optical coherence tomography (OCT) revealed a central pre-injection macula thickness of 445 µm on the right and 446 µm on the left with intraretinal and subretinal fluid (Figure 6). Post-injection central macula thickness was 299 µm with scarring on the right. On the left, central macula thickness was reduced to 232 µm.

At her 2-month clinic visit, she had no light perception and had developed cataracts with posterior synechiae which prevented fundal examination with intraocular pressures of 2 mmHg on the right eye and 3 mmHg on the left eye.

After extensive work-up and ophthalmological expert consultation, she was initiated on long-term albendazole at a dose of 600 mg daily orally. She was continued on her ART for the HIV infection.

She was provided with counselling by the social worker, referred to a society for the blind and a disability grant was initiated.

By the fifth month after presentation, her visual acuity remained unchanged with no light perception bilaterally.

## Discussion

In our case, the positive patient history of living on a farm, consuming meat and swimming in rivers, in conjunction with the clinical findings of panuveitis, investigations revealing eosinophilia and the echinococcus serology, points to a diagnosis of Primary Intraocular Echinococcus. Orbital hydatid cyst is found in developing countries; the diagnosis depends on the exposure in an endemic area supported by ultrasonography.^9^ Typical ultrasonography findings show cystic cavities along with a typical double-wall sign in the presence of hydatid sand attached to the walls.^9^ This was further supported by our findings of intraocular cystic ‘double-walled’ lesions on B-scans.

Echinococcosis, also called hydatid disease, is a common zoonotic parasitic disease occurring in various regions of the world.^10,11^

Echinococcosis occurs as a result of infection by larval stages of the cestodes *E. granulosis*.^12^ This typically involves a dog–sheep–dog cycle. *Echinococcus* eggs get transmitted in a cycle ‘hand-to-mouth’ via ingesting water or food (from soil contaminated with stool from infected dogs). The disease is prevalent in poor socioeconomic areas where people use dogs to tend their herds.^13^ This is found commonly in areas with poor sanitation where slaughtering of livestock is conducted without proper supervision.^14^

Isolated orbital hydatid disease without involvement of other organs is rare, which could be against our diagnosis as no other cystic lesions were found.^15^ Despite its rarity, it does question the possibility of another route of spread (i.e. the eye mucosa) or relate to an atypical presentation due to symptomatic HIV. In the orbit, the superolateral and superomedial areas are commonly affected.^16^ Many hydatid cysts are asymptomatic.^16^ Patients can present with painless nodular growth in the eyelid, or proptosis associated with periorbital pain and reduced ocular movements. It can present as impaired vision, headache, recurrent inflammation, keratitis, chemosis or proptosis.^16^ Our patient presented with progressive blindness with no overt proptosis.

Case reports on HIV and *Echinococcus* spp. co-infection are limited and have a vast geographical distribution.^2,3^ It is postulated that *Echinococcus* spp. facilitates the TH2 (T-helper) immune response and suppression of the CD4+ T-cell population in HIV patients.^17^ Thus far, no association has been found between a low CD4+ T-cell count and the probability of acquiring human echinococcosis.^3^ Our patient was found to have virological failure with low CD4 levels, which may or may not have contributed to her atypical presentation.

Atypical presentations of hydatid disease such as neuronal involvement and multifocal cysts are more common in HIV-infected individuals.^18,19^ Serological diagnosis of echinococcosis in HIV may be challenging. As a result of reduced antibody responses, serological testing is poor in HIV-infected individuals.^20,21^ A negative result does not rule out echinococcosis, and positive results should be carefully interpreted as there is a risk of underestimation.^3^

Hydatid cystic disease can be medically and/or surgically treated. Curative treatment including cystectomy and anthelminthic treatment can increase the CD4+ T-cell count causing an immune reconstitution syndrome (IRIS).^19^ IRIS following the treatment of echinococcosis in HIV co-infected patients is currently unknown. The medical therapy of choice is anthelminthics in the form of mebendazole or albendazole, which are the gold standard of care.^22^ This may cause an increased inflammation around the lesions. The use of corticosteroids can be used to diminish induced inflammation. Surgical removal may be required for isolated ocular lesions causing vision loss. Currently, there are no known treatment modalities for intraocular hydatid disease. To prevent recurrence, anthelminthic drugs and steroids have been advised after surgical removal.^23^

## Differential diagnosis

A differential diagnosis, contributing to vision loss, would include Toxic Optic Neuropathy (TON) secondary to anti-tuberculosis drug use. TON is defined by visual impairment due to optic nerve damage by a toxin.^24^ This condition often presents as painless, progressive, bilateral, symmetrical visual decline with variable optic nerve head pallor,^25^ which is a similar presentation of our case.

Anti-tuberculosis agents such as ethambutol and isoniazid may produce a TON. Ethambutol is implicated in 1%–5% of cases.^26^ Visual decline usually starts months after drug commencement, which was within the timeframe of our case presentation.^26^ Dyschromatopsia is a sign of toxicity^26^ and central scotomas, which are common;^27^ however, both were not seen in our patient. In addition, the findings of ‘double-walled cysts’ and eosinophilia are not reported in studies of TON, which can suggest an alternate diagnosis. Isoniazid TON may also be associated with bilateral optic disc swelling as seen in our patient at presentation.^27^ The cessation of drugs could improve vision loss; however, in our case, after anti-TB presentation there was a worsening of vision culminating in complete blindness. Paradoxical worsening after anti-TB drugs has been reported. The Jarisch-Herxheimer-like reaction has been described as the immune reconstitution occurs during treatment.^28^ The diagnosis of intraocular TB is most times only presumptive in this case without histological evidence. Uveitis is an early presentation in TB and can be considered in the differential diagnosis of any type of intraocular inflammation. Primary ocular disease, like in echinococcus, is rare. We also did not see any retinal lesions that may take the form of either focal tubercle or subretinal abscesses, which is common in TB.^29^

Despite negative serological markers, another differential of necrotizing herpetic retinitis complicated by retinal detachment can be considered in this case. Viral retinitis can present early on as anterior uveitis, retinal arteritis, a hyperaemic optic disc, inflammatory vitreous opacities and elevated intraocular pressures with clinical progression to retinal detachment, optic atrophy, and retinal vascular occlusion.^30^ HIV-infected individuals at lower CD4 counts are at greater risk of viral infections such as cytomegalovirus (CMV) infection, HSV and varicella zoster virus (VZV).

## Conclusion

Ocular echinococcosis, although rare, can lead to severe visual impairment. Currently, there are no known treatment modalities for intraocular hydatid disease. Case reports on HIV and *Echinococcus* co-infection are limited with a potential gap for research. An important differential diagnosis in our case, due to her prior exposure to anti-TB therapy, was that of TON. Our case report highlights hydatid disease as an uncommon, but important consideration in HIV-associated vision loss.
